# Fabrication of Fresnel plates on optical fibres by FIB milling for optical trapping, manipulation and detection of single cells

**DOI:** 10.1038/s41598-017-04490-2

**Published:** 2017-06-30

**Authors:** Rita S. Rodrigues Ribeiro, Pabitra Dahal, Ariel Guerreiro, Pedro A. S. Jorge, Jaime Viegas

**Affiliations:** 1 INESC TEC, Rua do Campo Alegre, 687, Porto, Portugal and Departamento de Física e Astronomia, Faculdade de Ciências, Universidade do Porto, Rua Campo Alegre, 687 Porto, Portugal; 2 Masdar Institute of Science and Technology, PO BOX 54224 Abu Dhabi, United Arab Emirates

## Abstract

The development of economical optical devices with a reduced footprint foreseeing manipulation, sorting and detection of single cells and other micro particles have been encouraged by cellular biology requirements. Nonetheless, researchers are still ambitious for advances in this field. This paper presents Fresnel zone and phase plates fabricated on mode expanded optical fibres for optical trapping. The diffractive structures were fabricated using focused ion beam milling. The zone plates presented in this work have focal distance of ~5 µm, while the focal distance of the phase plates is ~10 µm. The phase plates are implemented in an optical trapping configuration, and 2D manipulation and detection of 8 µm PMMA beads and yeast cells is reported. This enables new applications for optical trapping setups based on diffractive optical elements on optical fibre tips, where feedback systems can be integrated to automatically detect, manipulate and sort cells.

## Introduction

Light driven tools, such as, optical tweezers (OTs), are one of the main breakthroughs of the last decades. Optical manipulation was first demonstrated by A. Ashkin, in 1970, where a micro particle was trapped by two counter propagating laser beams due to radiation pressure^1^.

Optical tweezers are regularly used in the immobilization and manipulation of a wide range of particles, while precisely measuring positions and applied forces. In microrheology, OTs play an important role in the measurement of viscosity of fluids using trapped beads as test probes^2^. In particle physics, the generation of cold atoms is attained due to magneto-optical traps^3, 4^, enabling further advances in quantum technology^5^. At the same time, biomedicine is equally benefiting from recent advances on OTs, with a diversity of applications being reported, such as research on red blood cell deformation and aggregation^6–8^, dynamics of molecular motors^9^, such as myosin and kinesin^10, 11^, or evaluation of forces generated by the transcription of enzymes in DNA strands^12^. Simultaneously, cell separation and sorting using optical trapping systems are also tools frequently used in biology with growing applicability^13^.

The platforms employed in the analysis of the particle targets normally rely on modified microscopes with high numerical aperture objectives, usually adapted to accommodate other parts, such as, spatial light modulators or quadrant photo detectors, for the generation of spatial traps and particle displacement monitoring^14^, respectively.

Nonetheless, the development of OTs is growing towards optofluidic platforms, envisioning lab-on-a-chip tools. The lenses responsible for the optical trapping are one of the key elements that may be improved. With this in mind, this paper presents a set of Fresnel zone and phase plates, fabricated on common optical fibres using focused ion beam milling for trapping purposes. The literature on trapping using single optical fibres has seen advances in the design and capabilities of the lenses^15^. Early on, optical fibre probes were limited to tapered fibres^16^, fabricated by chemical etching^17^, thermal processes^18^, among others^19^. For instance, Baojun Li has reported the use of tapered fibres for trapping, and manipulation of single or multiple targets^20, 21^. The vast range of applications of these fibre probes show their flexibility^22–24^. However, progress in microfabrication technology has enabled better-quality trapping probes, envisaging higher trapping control^25, 26^. FIB milling is a fabrication method that allows to control, with optical subwavelength resolution, the features of the designed structures, contrarily to the methods mentioned above. At the same time, besides trapping and manipulation, the structures presented in this work are also used for size-based detection of particles.

In this work, the choice of Fresnel diffractive structures is linked with three main reasons: Fresnel lenses are an alternative to conventional objectives, since their design allows tailoring features like focusing distance and numerical aperture, matching the desired values necessary in OTs; the planar design offers significant chances to be implemented in microfluidic channels^27, 28^, incorporated in optofluidic devices; and finally, the possibility to replicate these structures using nanoimprinting lithography, to fabricate a large number of fibre probes^29^. There is some literature reporting the fabrication of Fresnel plates on optical fiber tips using femtosecond laser micromachining and FIB milling. The first method was employed in the fabrication of structures mainly focused on light coupling devices^30–32^. Nevertheless, the second process was used to fabricate a structure designed for optical trapping of subwavelength particles^33^, on the contrary, the present work reports trapping, manipulation and detection of micron-sized particles and cells, foreseeing applications to larger target sizes, such as mammalian cells.

### Fabrication and optical characterization

#### Design

A Fresnel zone plate (FZP), is a diffractive optical element, composed by a sequence of concentric alternating opaque and transparent zones, with axial symmetry^34^. The radii of the successive rings are given by:

1 $${r}_{n}=\sqrt{n\lambda f+\frac{{n}^{2}{\lambda }^{2}}{4}},$$

where *n* is an integer, *λ* is the wavelength and *f* is the focal length. For *λ* ≪ *f*, Eq. (1) reduces to:

2 $${r}_{n}=\sqrt{n\lambda f.}$$

**Figure 1:**
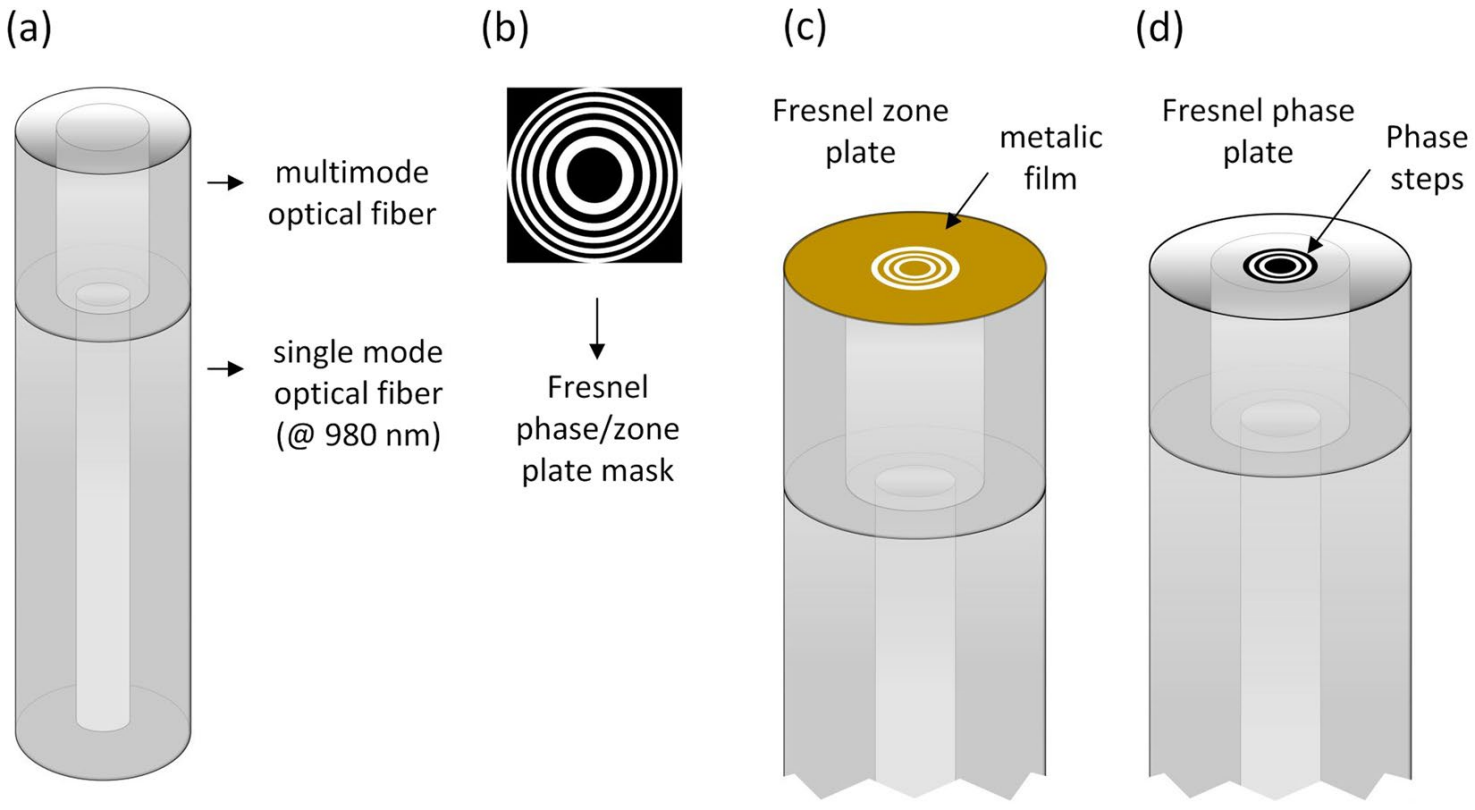
(**a**) optical fibre probe design composed by the SM fibre and the MM segment; (**b**) Fresnel phase/zone plate characteristic ring structure; (**c**) Fresnel zone plate on the tip of the optical fibre (coated with 20 nm Pt film); (**d**) Fresnel phase plate on the tip of the optical fibre (no film).

The intensity of the focal point is proportional to the number of zones. These structures have chromatic aberration, such as convex lenses, since *f* is inversely proportional to *λ*. In contrast, Fresnel plates have multiple foci: composed by the main focal point, *f*, and higher order focal points at *f/3*, *f/5*, …, with decreasing brightness^35^. From this, a Fresnel zone plate is basically an amplitude device. Nevertheless, an alternative plate may be achieved if the opaque zones are replaced by transparent π-phase steps. In this case the resulting focal spot will be brighter, increasing the optical conversion efficiency of the plate^36^, since the light is no longer blocked. This is the so-called Fresnel phase plate (FPP). The depth (*d*) of the phase zones is given by

3 $$d=\frac{\lambda }{2({n}_{plate}-{n}_{medium})},$$

where *n*_*plate*_ and *n*_*medium*_ are the refractive index of the plate and the surrounding medium, respectively. The size of FZP and FPP on single mode optical fibres (SMF) is limited by their mode field diameter, which is normally a few micrometres wide. Since the number of zones directly affects the intensity and width of the central peak of the diffraction pattern, it makes sense that mode-expanded optical fibres are used^30^. A scheme illustrating the optical fibre tips is visible in Fig. 1(a). The single mode fibre is spliced to a multimode one, allowing the Gaussian mode that propagates in the SMF to expand in the multimode segment. This results in a broader effective working area on the top of the tip. The cross section that is covered by the beam depends of the length of the MMF, and can be calculated through the numerical aperture relation.

#### Fabrication

The production of diffractive structures, such as Fresnel plates (see Fig. 1(b)), requires high resolution fabrication methods. To address this challenge, several authors have proposed the use of distinct processes, such as: femtosecond pulsed laser micromachining^30, 31^, e-beam lithography^27^, focused ion beam milling^37–39^, among others^28, 40^. In this work, the fabrication of the Fresnel zone and phase plates is carried out using focused ion beam (FIB) milling^41, 42^.

The single and multi-mode fibres described in this paper are SM 980 (Thorlabs) and AFS 50/125Y (Thorlabs), respectively. The procedure starts with the splice of a SM with a MM fibre segment. After this, the MMF is cleaved at an optimal length. A USB camera with a magnifying lens is placed above the cleaving machine allowing the observer to control the process. The fibre is moved, relatively to the blade, using a micrometric positioner placed before the machine. The imaging setup allows a continuous magnification range from 50x to 500x, and the micro stage has a resolution of 0.5 µm thus enabling to cut the MMF with enough accuracy. The length of the MM is important because it determines the dimensions of the fibre cross section covered by the beam. In this case, the extent of the MMF is optimized so that the beam covers the diameter of the plate that will be milled on the fibre tip. Once cleaved and cleaned, the fibres are finally mounted in a 45° stub, that is suitable to be used in the FIB.

The FIB is a nano resolution fabrication process, where Gallium ions are accelerated towards the sample^43^. Depending on the acceleration current, it can either be used in the removal of atoms from the sample’s surface, or to image it. In this case the FIB is integrated in a dual-beam system with a scanning electron microscope (Quanta 3D, FEI Company), providing high resolution imaging.

In the fabrication of the FZP, initially a layer of 20 nm of Platinum (Pt) is deposited over the optical fibres, using e-beam evaporation. The metallic film is necessary for two reasons: to avoid charging effects on the dielectric fibre surface during the FIB milling and to block the light, by the odd zones of the plate (Fig. 1(c)). In contrast, for the fabrication of FPP, the optical fibres are coated with a thin layer, 5 nm, of Gold/Palladium (Au/Pd) (see Fig. 1(d)). In this case, the goal of this layer is merely to avoid the charging effects during the milling and will be removed after the fabrication of the phase plates. In this paper, the fabrication of four different types of plates is described: two FZP (FZP-1 and FZP-2) and two FPP (FPP-1 and FPP-2).

**Figure 2:**
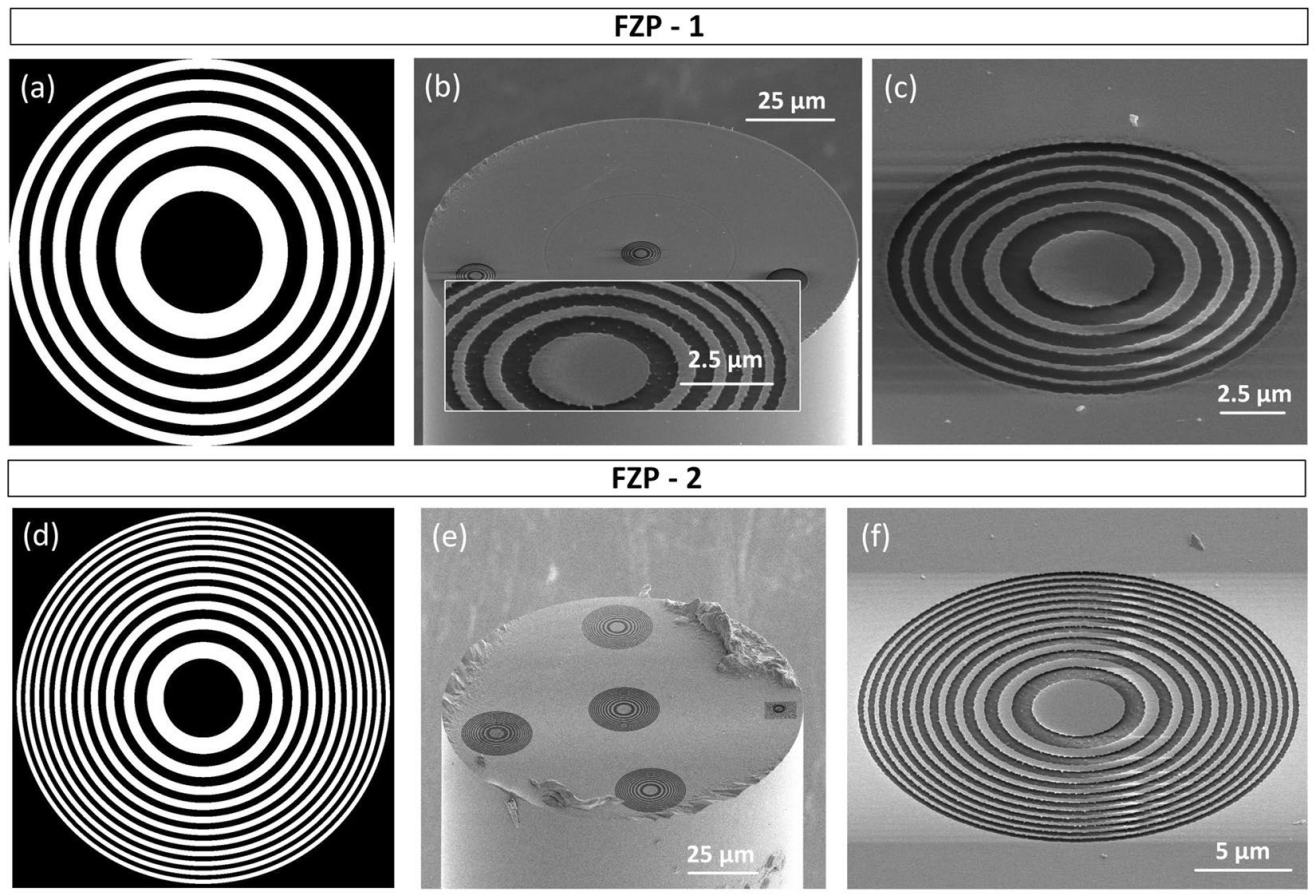
Fresnel zone plates: (**a**) amplitude mask used to fabricate FZP-1; (**b**) image of the optical fibre with FZP-1, (inset) zoom of a FZP fabricated with a metallic film of Au/Pd; (**c**) zoom of FZP-1; (**d**) amplitude mask used to fabricate FZP-2; (**e**) image of the optical fibre with FZP-2; (**f**) zoom of FZP-2.

FZP-1, composed by 10 zones, is projected to have a focal distance of 3 µm and a radius (r_10_) of 5.42 µm. The amplitude mask used during the fabrication process is shown in Fig. 2(a). During the fabrication, the exposure dose is controlled by the dwell time, corresponding to a bitmap pixel value (Fig. 2(a)). In this fashion, for a white pixel (level 255) the dwell time was set to 2 µs, while for a black pixel (level 0) the beam is blanked. The ion beam current was set to 0.3 nA (beam diameter 31 nm), corresponding to a milling time of approximately 41 seconds. An image of the optical fibre with the FZP can be seen in Fig. 2(b), corresponding zoom in Fig. 2(c). Analysing this image, a radius of 5.57 µm is measured. This value is slightly larger than the projected one, having a relative error of 2.73%.

The second amplitude plate, FZP-2 is composed by 22 zones, and is dimensioned to have a focal distance of 6 µm, corresponding to a radius (r_22_) of 11.37 µm. The amplitude mask correspondent to the profile imprinted on the top of the fibre is presented in Fig. 2(d). The beam current and dwell time were 0.3 nA and 1 µs, respectively. The overall milling time was approximately 4 minutes. The resulting zone plate can be seen in Fig. 2(e,f). From the images, a radius (r_22_) of 12.18 µm was estimated, with a relative error of 7.05%. Please note that the structures visible in the boarder or the fibre are just for control of the fabrication conditions, the performance of the central plate is not influenced by those.

Previous to the fabrication of FZP-1 and 2, some tests were carried out, to check the final conditions of the plates surfaces, regarding the smoothness. When using Au/Pd to cover the optical fibres, after the milling, the surface was quite rough. This is due to the different sputtering rates for each type of metal. An example of a zone plate with an irregular surface is visible in the inset of Fig. 2(b). In this regard, the fibres were covered by Pt.

**Figure 3:**
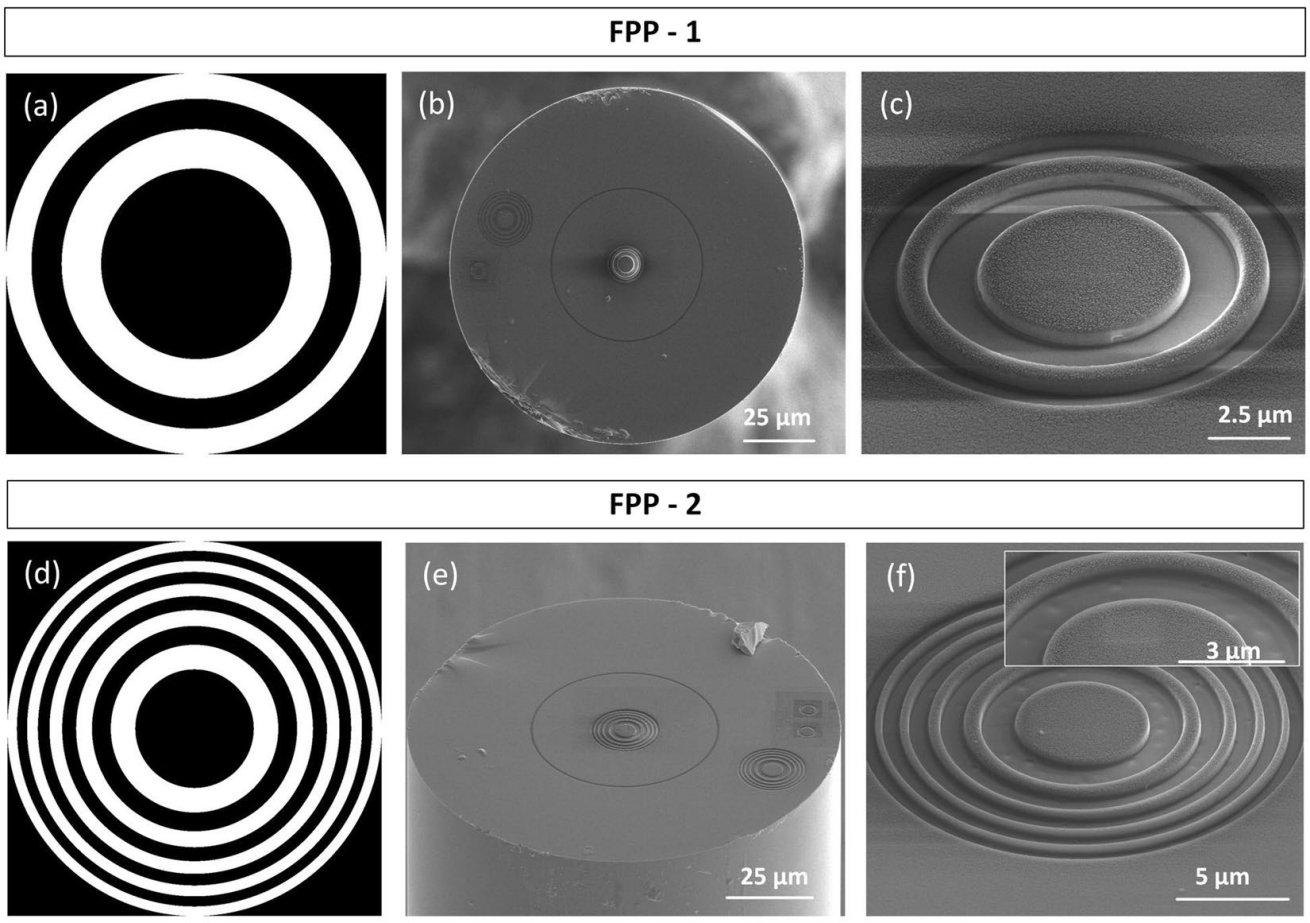
Fresnel phase plates: (**a**) amplitude mask used to fabricate FPP-1; (**b**) image of the optical fibre with FPP-1; (**c**) zoom of FPP-1; (**d**) amplitude mask used to fabricate FPP-2; (**e**) image of the optical fibre with FPP-2; (**f**) zoom of FPP-2, (inset) details of the first three zones of FPP-2.

FPP-1 is composed by 4 zones, of alternated depths. It was adjusted to have a focal distance of 10 µm, corresponding to a calculated radius (r_4_) of 6.26 µm. The optical fibre tip used in this case, has a MM segment with a length of ~30 µm. Figure 3(a) depicts the amplitude mask used in this particular case. The beam current and dwell time were 0.1 nA and 1 µs, respectively. The overall milling time was approximately 15 minutes. The resultant plate can be seen in Fig. 3(b,c). Once again, the radius of the fabricated plate (r_4_ = 6.11 µm, relative error = 2.35%) was slightly different from the projected one. From the SEM images, the height of the phase discontinuities is estimated to be around 534 nm. After the FIB milling, the optical fibres were cleaned with *aqua regia*, to remove the metallic film.

In order to verify the influence of the number of rings a second zone plate is here presented. FPP-2 is composed by 10 zones, and is also dimensioned to have a focal distance of 10 µm, corresponding to a calculated radius (r_10_) of 9.90 µm. The length of the MM segment is ~50 µm. Figure 3(d) depicts the amplitude mask used in this particular case. The beam current and dwell time were 0.1 nA and 1 µs, respectively. The overall milling time was approximately 18 minutes. The phase plate can be seen in Fig. 3(e,f). The radius was estimated to be 10.14 µm with a relative error of 2.43%. The height of the phase steps is 419 nm. The inset of Fig. 3(f) shows some imperfections on the surface of the plate, due to some constrains of the FIB gun. However, the dimensions are very reduced (Fig. 3(f), inset), and no effects were observed on the output beam profile.

#### Characterization of Fresnel plates

**Figure 4:**
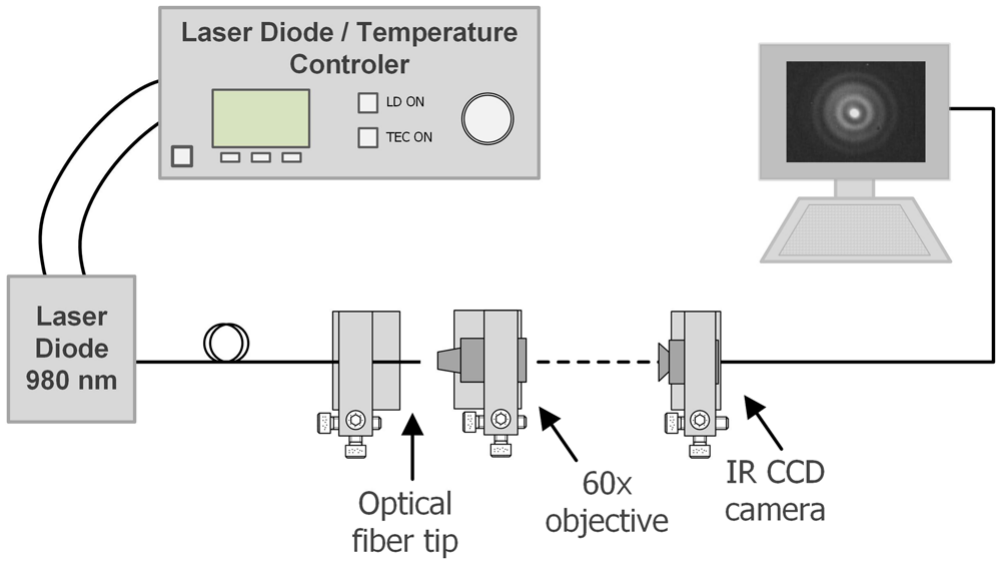
Experimental setup used to analyse the output beam from the optical fibre tips with the Fresnel zone and phase plates.

The characterization of the output beams generated by the Fresnel zone and phase plates was performed using the setup presented in Fig. 4. First, the optical fibre tips were spliced to a pigtailed laser source (Lumics, 980 nm, 500 mW). Then, they were placed in a micromanipulation stage, which can be moved horizontally. This allows to acquire images of the optical beam at different distances from the optical fibre, since the beam is projected into a CMOS camera using a 60x objective.

**Figure 5:**
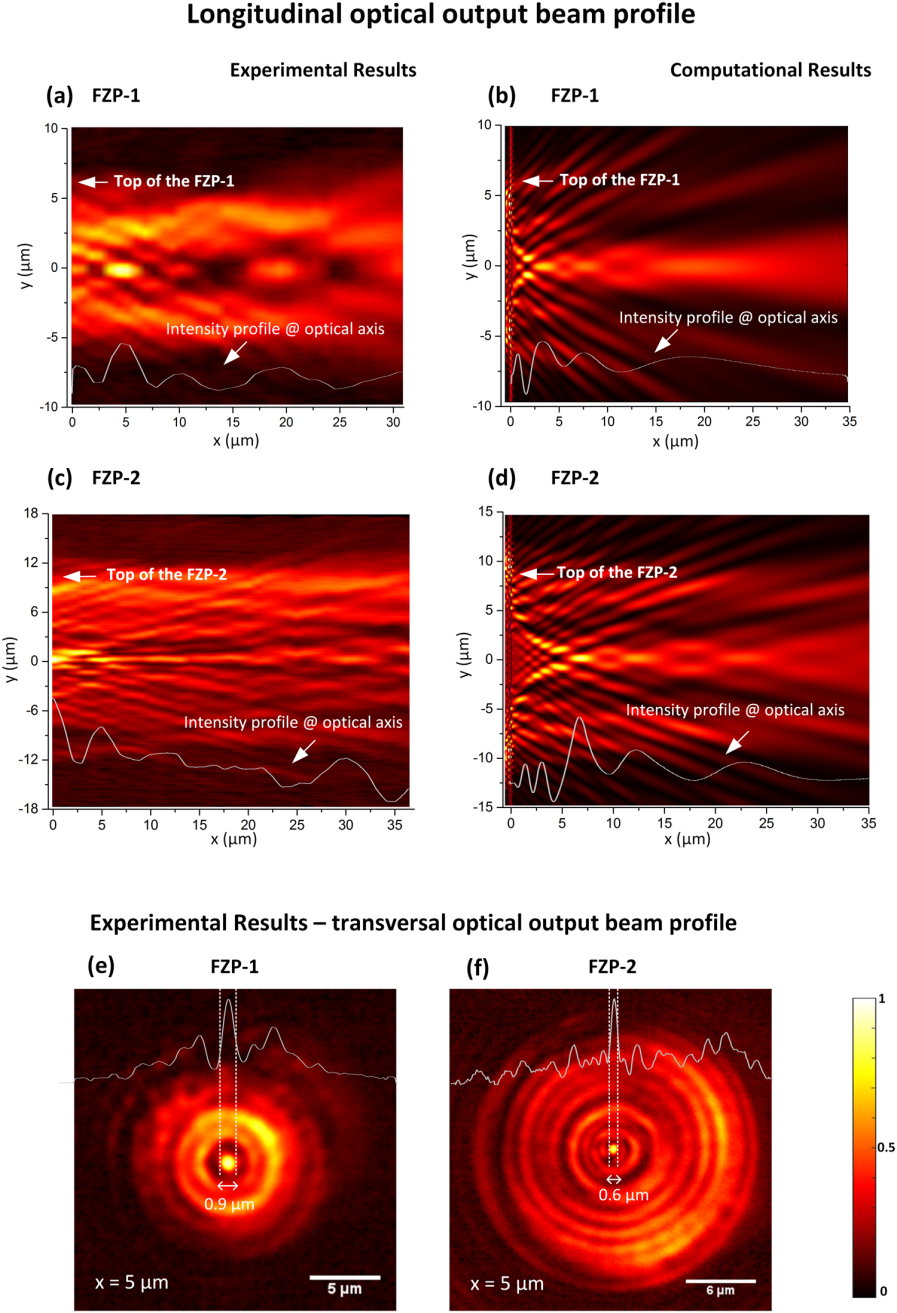
Longitudinal optical output beam profile for FZP-1: (**a**) experimental; (**b**) computational. Longitudinal optical output beam profile for FZP-2: (**c**) experimental; (**d**) computational. Transversal optical output beam profile at the main focal point: (**e**) FZP-1; (**f**) FZP-2.

The structure of the FZP-1 output optical beam is presented in Fig. 5(a). Sequential cross sections (*yz*) were first acquired at different distances (along *x*), using the setup presented in Fig. 4. Then, using ImageJ software^44^, the pictures were combined into a 3D view, allowing to have the orthogonal profile (*yx*) shown in Fig. 5(a). With the purpose of investigating the resulting features of the structures, computational simulations mimicking the fabricated plates were performed. The electromagnetic system was modelled based on the implementation of the finite difference time domain method, MEEP^45, 46^. A 2D representation of the optical fibre was selected, considering the cylindrical symmetry of the system, and to avoid time consuming simulations. The refractive index of the optical fibre and the surrounding media were set to 1.458 and 1.000, respectively, while the source wavelength was 980 nm. The metallic structure was mimicked by a thin layer with a high refractive index, to block the light in these regions. In this particular case, the profile obtained in Fig. 5(b) was computed. Observing both, computational and experimental representations of the output electric intensity, the focal spot is located at 3 µm and at 5 µm, respectively. This is demonstrated by the plot of the intensity curves, along the propagation direction, in Fig. 5(a,b). Besides the main focal point, the plate exhibits two secondary focusing positions, at 10 µm and 20 µm (experimental values). The number of extra focal points is supported by the simulations, although the locations are not exactly the same.

To analyse the second zone plate, FZP-2, an analogous process was followed. Hereof, Fig. 5(c,d) depicts the experimental and computational results, respectively. In this case, FZP-2 was dimensioned to have a focal distance of 6 µm, which is verified by the simulations. Regarding the experiments, the focal distance is situated at 5 µm. Similarly to FZP-1, this structure also causes secondary focal points, which are also corroborated by the simulations.

In Fig. 5(e,f), the experimental transversal profiles at the main focal points for each zone plate are depicted. In this case, this allows to calculate the full width at half maximum (FWHM) of the central peak of the resultant patterns. For FZP-1, the inner peak has a dimeter of 0.9 µm, whereas for FZP-2 is 0.6 µm. This shows the influence of the number of zones, i.e., although the higher focal distance of FZP-2, the focusing of the light is stronger, since the plate is composed by twice the number of diffractive rings.

**Figure 6:**
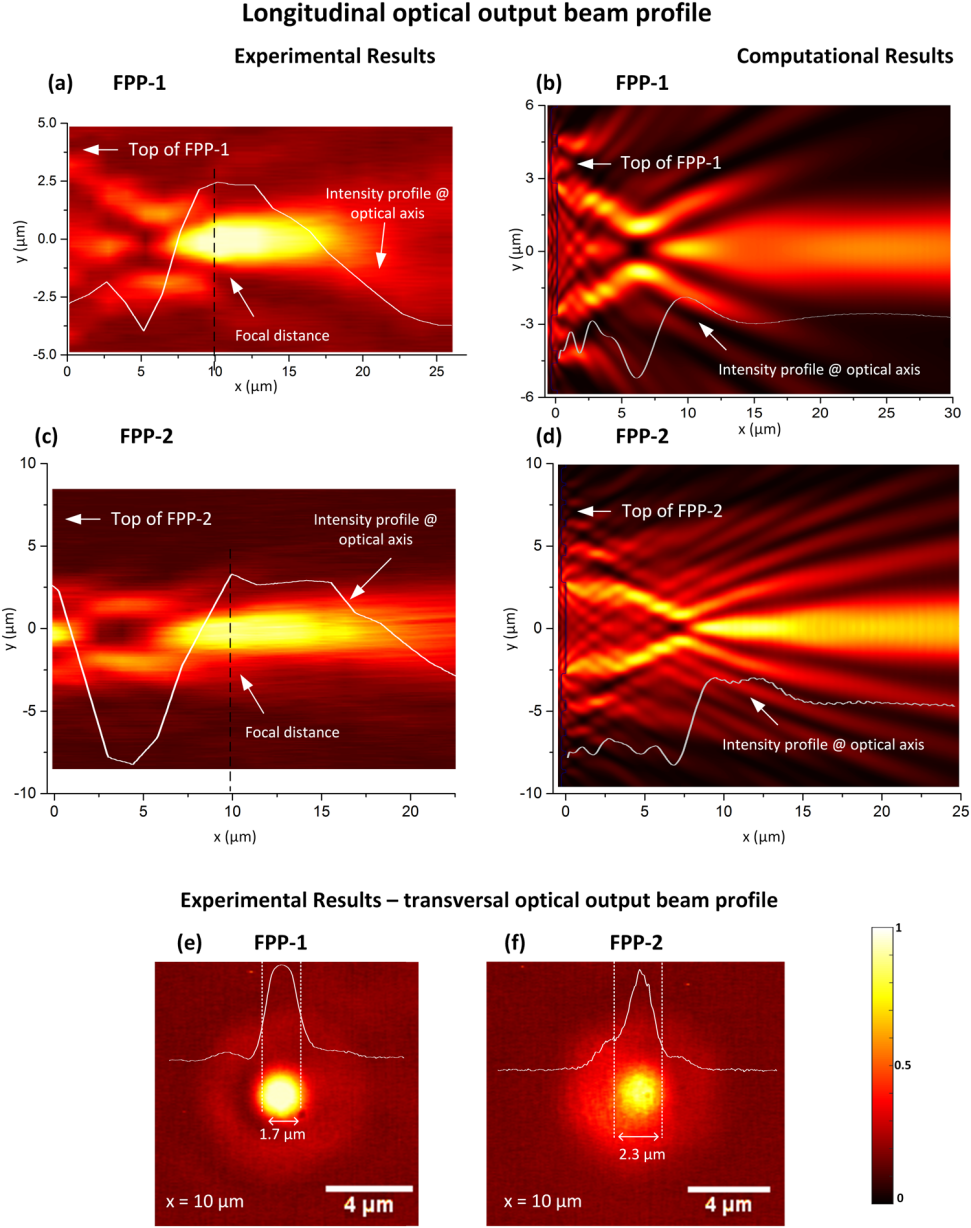
Longitudinal optical output beam profile for FPP-1: (**a**) experimental; (**b**) computational. Longitudinal optical output beam profile for FPP-2: (**c**) experimental; (**d**) computational. Transversal optical output beam profile at the main focal point: (**e**) FPP-1; (**f**) FPP-2.

The analysis of the output beams of the phase plates (FPP-1 and FPP-2) follows a similar approach. Hereupon, Fig. 6(a,b) depict the results for FPP-1, which is projected to have a focal distance of 10 µm. Overall, the experimental and computational results match with the projected value. Looking at the measured intensity profile plotted in Fig. 6(a), the maximum of intensity should be located at ~10 µm. In this regard, this is in line with the computational results, Fig. 6(b), where the intensity maximum is located at 10 µm, and slightly decreases after this point.

Figure 6(c,d) depict the results for FPP-2, which was also projected to have a focal point at 10 µm, however, containing more rings than the previous structure. Once again, from the experimental results (Fig. 6(c)) one may conclude that the maximum of intensity is located at ~10 µm. The simulations also present a maximum at ~10 µm, that slowly loses intensity along a few micrometres. This results are coherent with the previous ones, presented for FPP-1.

Figures 6(e,f) show the transversal profiles experimentally acquired at the maximum intensity positions for each phase plate. The FWHM of the central peak for FPP-1 is ~1.7 µm and for FPP-2 is ~2.3 µm. In this case, increasing the number of diffractive rings caused a slightly broader peak.

## Discussion

The studies of the optical properties of the zone and phase plates presented in the previous sections demonstrated some important features that differentiates both Fresnel diffractive structures. Table 1 summarizes some of the measured parameters. The zone plates cause a narrower central peak at the focal point, however, present two extra focal points in addition to the main one. In contrast to the zone plates, the phase plates only originate one focal point, nevertheless, the central peak is wider in the transversal direction. The efficiency of the devices was calculated based on the ratio between the power detected at the output of a cleaved optical fibre and the output detected at the output of each device. The measured values were: 33% and 38% for the FZP-1 and 2 and 60% and 67% for FPP-1 and 2, respectively. This proves that the losses are higher for the case of the zone plates, since the light is blocked by alternated zones, while the phase plates are more efficient in the conversion of the optical profiles.

**Table 1:** Fresnel Plates parameters (distances in µm).

Fresnel Plate	N	f (theo.)	r_N_ (theo.)	r_N_ (exp.)	E(r_N_)	f (simul.)	f (exp.)
FZP-1	10	3	5.42	5.57	2.73%	3	5
FZP-2	22	6	11.37	12.18	7.05%	6	5
FPP-1	4	10	6.26	6.11	2.35%	10	10
FPP-2	10	10	9.90	10.14	2.43%	10	~10

Besides this, a general comment concerning the differences between the computational and experimental optical field profiles should be done. Looking at Figs 5(a,c) and 6(a,c), the fabricated tips generate an output optical field that maintains some degree of collimation, while in the simulations (Figs 5(b,d) and 6(b,d)) it is more evident that the resulting output beams diverge. Although the simulated structures have been dimensioned to reproduce the fabricated ones, there are always some parameters that may deviate from the intended ones. For instance, the fabricated structures may lack absolute circular symmetry, causing an impact on the structure of the output beam. Furthermore, during the scan of the ion beam, the milling is more pronounced in the edges that in the centre, inducing a lensing effect^41^. On the other hand, during the fabrication there is also a possibility to have Gallium ions implanted on the fibre surface, as well as carbon residues, due to the contamination of the FIB chamber. These can be responsible for blockage or absorption of the light, as addressed by Janeiro *et al*.^42^. In the particular case of the zone plates, the simulations have taken into account the dominant effect of the structure produced by the pattern on the fiber tip, but have left out second order corrections. These are associated with the dispersion of the metal and the possibility of producing localized surface plasmons that might result in a small contribution of the spatial distribution of light of the output beam. Although most of the electric field is parallel to the surface of the metal rings, and thus is not effective in producing surface plasmons, at the edges of the rings it is indeed possible to have plasmonic excitation, which changes the effective dimensions of the rings and introduces a minor correction in the spatial distribution of the beam structure. As a result, some spatial components of the beam dispersed by the metallic structure may gain slightly different phase delays than predicted by the simulations, thus justifying the contrast and definition between simulations and experimental results. However, including more realistic parameters at such level would add to much complexity to the model, which at present was simply intended to guide the design. Nevertheless, these properties will be explored in futures works as the plasmonic features may provide interesting mechanisms for further focusing and sensing abilities.

Overall, from the comparison of the two types of devices it can be said that FPP are definitely more easily fabricated to fit a simple modeling approach providing interesting features as micromanipulation devices. Instead, the FZP show a more complex behavior, that requires a more complex modeling approach to explore its full potential.

In the following section, FPP-1 will be tested for optical trapping of dielectric particles and yeast cells. On the one hand, the match between the computational and experimental results, regarding Fresnel phase plates, proves that the methodology presented in this work is adequate and trustworthy. Besides this, the phase plates have better optical conversion efficiency than the zone plates. On the other hand, the zone plates experimental results differ from the projections, due to the facts above mentioned: implantation of Ga ions, defects on the ring size and possible excitation of plasmons. At the same time, the zone plates are also covered with a metallic film, which might act as a source of heating, that can compromise the trapping effect, and the sample integrity. In this regard, choosing a phase plate not only fits the need for optical trapping but also represents less constrains from the fabrication to the application. Regarding the choice of FPP-1 rather than FPP-2, this relies on the dimension of the central peak, which is narrower at the focal distance, contributing to an increase of the optical force responsible for the trapping.

### Optical trapping using Fresnel plates

#### Manipulation setup

**Figure 7:**
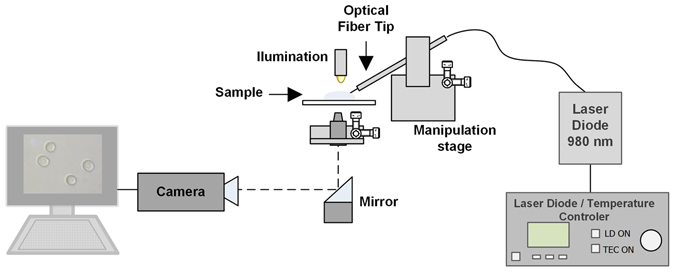
Optical manipulation setup.

To test the Fresnel plates for optical trapping, the setup depicted in Fig. 7 was used. This is composed by two main parts: an image acquisition system and a 4-axis motorized micromanipulator to precisely handle the optical probes. The imaging system is essentially an inverted microscope, composed by a CMOS camera (EO-2018C, Edmund Optics) and an objective. To assemble the optical probes at the stage, first they were spliced to a 980 nm pigtailed laser diode (500 mW, Lumics) and then carefully inserted into a metallic capillary. After this, the capillary was attached to the stage and adjusted at a suitable angle. Then, the fibre was inserted into the sample that was placed over the glass slide.

### Optical trapping of PMMA particles

**Figure 8:**
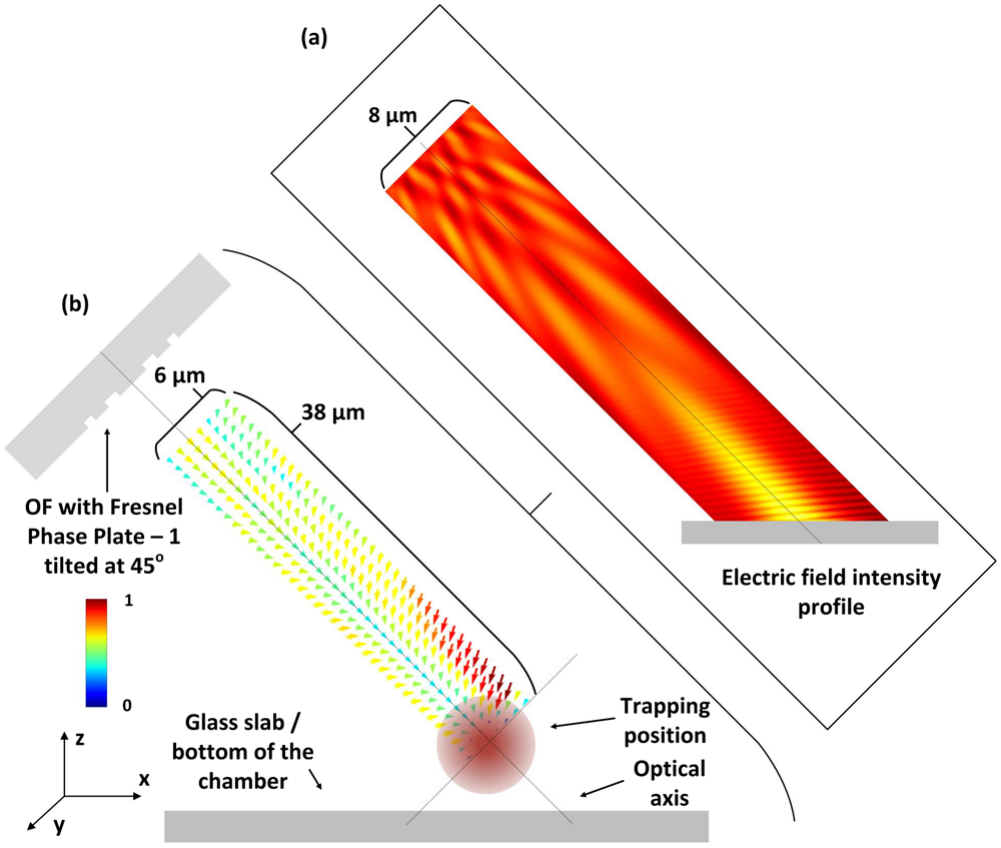
(**a**) Field intensity profile for FPP-1 tilted at 45° immersed in water; (**b**) corresponding force distribution map, for the particular case of 8 µm PMMA beads.

To understand how the optical beam is affected by the media, and how do the optical forces act, some simulations based on the FDTD method, earlier mentioned, were performed. The fibre was modelled using the experimental features of FPP-1, nevertheless, the refractive index of the media was set to 1.32 (instead of 1,00), since the experiments are done in an aqueous medium. The fibre was tilted at 45°, and a glass slide was included in the computational configuration, to better reproduce the experimental conditions. Figure 8(a) represents the electric field intensity, simulated in the mentioned conditions. Generally speaking, the focal distance increased, being located near the glass slab surface, and the beam spot became wider. This is caused by the increase in the solution refractive index to 1.32, resulting in a reduced contrast and lower focusing power. The optical forces acting on the PMMA particles are calculated based on the Lorentz model, considering the beads composed by dipoles^15, 47^. Thus, the force is given by:

4 $$\overrightarrow{f}=\frac{1}{2}\,{\varepsilon }_{0}({\varepsilon }_{p}-{\varepsilon }_{m})\nabla {\rm{I}}$$

where *ε*_0_ is the vacuum permittivity, *ε*_*p*_ is the particle relative permittivity, *ε*_*m*_ is the relative permittivity of the surrounding media and **I** is the electric field intensity. The calculations of the optical forces are performed for different positions of the particle, allowing to obtain a map with the distribution of the net forces, as depicted in Fig. 8(b). The available results show that when the bead is located aside the stable trapping position, it is first driven to the optical axis, and then towards the optical trap, close to the glass slab. Consequently, the expected trapping position is no longer at 10 µm from the optical fibre, but at the new focal position, as shown by the simulations, and corroborated by the following experiments.

**Figure 9:**
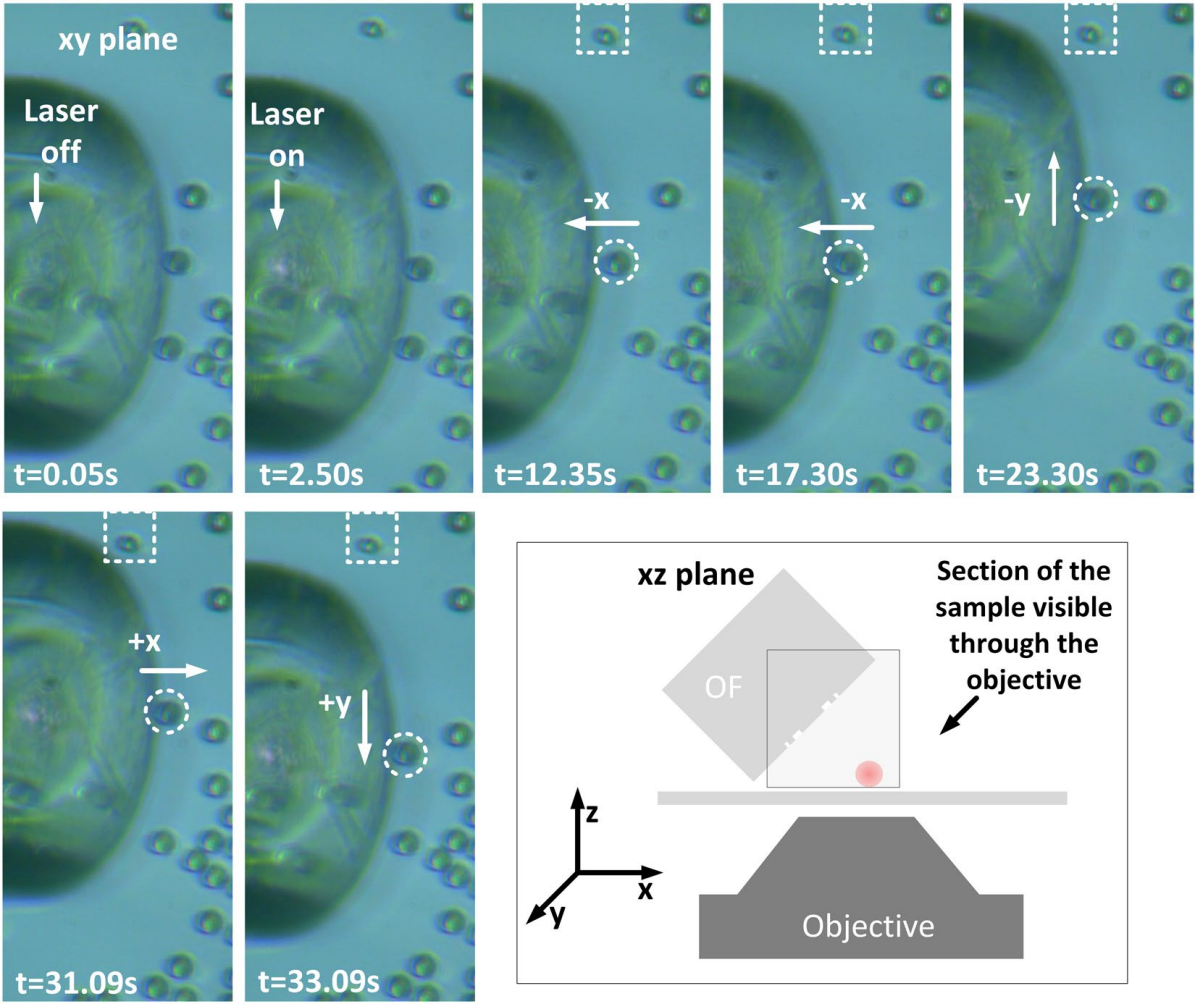
Demonstration of optical trapping of a PMMA particle in the xy plane. Scheme depicting the observer view, to demonstrate that the optical fibre does not touch the trapped particle (see Supplementary Video 1).

The optical fibre probe, containing FPP-1, was assembled accordingly to the procedure previously described, with an inclination angle of 45°. After this, a drop of an aqueous sample containing 8 µm diameter PMMA beads (1.4843 refractive index^48^) was placed on the glass slide. Using the motorized micromanipulator, the fibre was then immersed in the sample. Through the imaging system, the fibre was adjusted to be close to the glass slide, and when in the vicinity of some PMMA beads, the trapping was tested. Please see Fig. 9, and Supplementary Video 1. At t = 0.05 s the laser was off, and the fibre was positioned so that a bead was very close to the trapping point found in the computational simulations. After this, the laser was turned on (2.50 s) and the fibre was moved in the −x direction (12.35 s). The frame acquired at t = 17.30 s shows that the bead (within the white circle) was moved towards the trapping position, while the particle delimited by the white square remained in the same position since the beginning of the experiment. This was followed by the displacement of the fibre in the −y direction (23.30 s), and simultaneous movement of the particle. The probe was lastly moved in the +x (31.09 s) and +y (33.09 s) directions driving the particle with it. This demonstrates that the phase plate is able to trap and move PMMA particles in 2-dimensions, i.e., the *xy* plane. To make the actual probe configuration more clear to the reader, close attention should be paid to the scheme in Fig. 9, where it becomes evident that the fibre does not touch the beads during the trapping process. In the acquired frames the bead and the upper border of the fibre are superposed in the image but are located at different planes, explaining the defocusing of the fibre, while the particle is focused.

**Figure 10:**
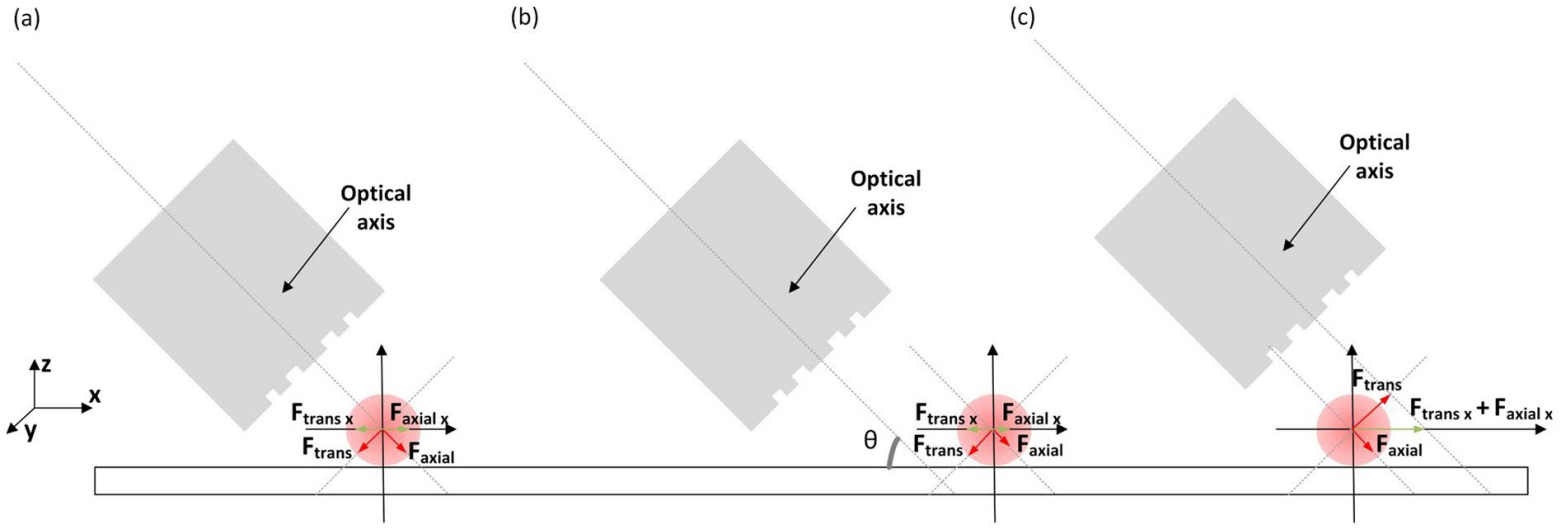
Optical forces acting on the particle, when it is at different locations: (**a**) at the trapping position F_trans x_ and F_axial x_ balance in the xy plan; (**b**) after the trapping point F_trans x_ exceeds F_axial x_ moving the particle towards the stable point; (**c**) before the trapping point, both F_trans x_ and F_axial x_ contribute positively to drive the particle to the stable position.

The optical forces acting on the particle can be described by the scheme depicted in Fig. 10. Since the fibre is tilted, the optical axis does not match the *x* direction in the *xyz* referential. Instead, it is rotated by θ. Consider Fig. 10(a) where the particle is located nearby the trapping point. In this case, the particle feels two forces, the axial and the transversal force. When they balance with each other, the particle is stably trapped. Otherwise, if the particle is beyond the trapping position, Fig. 10(b), the transversal force acts as a restoring force, and the particle is again driven to the trap. At last, when the bead is before the trap, the transversal and axial forces will positively contribute to guide the particle towards the steady position. These components of the force contribute to a stable trap in the *xy* plane, corresponding to a 2D trapping. In the meantime, in the *z* direction, the particle is confined in the vicinity of the glass slab. To take this into account, the revised Stokes equation, that is, Faxen’s law, is used to calculate the optical forces in the *xy* plane^14^. This form of the drag equation considers the particle close to a boundary, in this case, the glass slab. The total optical force acting on the particle is given by the sum of the inertial force (1^st^ term) and the drag force (2^nd^ term):

5 $${F}_{opt}=F+{F}_{drag}=m\frac{{\partial }^{2}s}{\partial {t}^{2}}+6\pi \eta r\varsigma \frac{\partial s}{\partial t}$$

where η is the viscosity of the medium, *r* is the radius of the particle, *ς* is a correction factor that considers the proximity of the particle to the cover glass surface taking the value of 3.084 for a gap nearby zero, and *s(t)* denotes the trajectory of the particle. This is decomposed into the *x* and *y* component for the 2D case. The inertial force is not considered since the Reynolds number is very small for micrometric particles. Consequently, when the particle is stably trapped, the optical force has to be strong enough to balance with the drag force. The dynamics of the particle in the trap was studied following the next procedure. First a bead was trapped, then the laser was turned off, and the fibre was moved. After this, the laser was again turned on, and since the fibre is only a few micrometres away from the bead, it was attracted towards the stable position. This was performed several times for each direction, −x, +x, −y and +y and the process was repeated for a range of powers with the fibre tilted at 45° and 30°. Using a particle tracking software from ImageJ^49^, the trajectories of the particles were attained. Whit this information the position versus time graphs of the trajectories were plotted. Doing a fit to this graphs and employing the first derivative, the optical forces were calculated according to Eq. 5.

**Figure 11:**
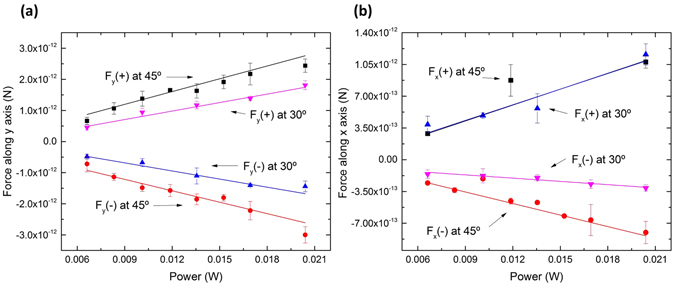
(**a**) Force along the *y* axis, measured with the optical fibre positioned at 45° and 30°; (**b**) Force along the *x* axis, measured with the optical fibre positioned at 45° and 30°.

The plots presented in Fig. 11 show the calculated values for the optical force along the *y* and *x* directions considering the particle tilted at 30° and 45°. As expected, the net optical force increases with the power, and as commonly measured, the values are within the pN range. Also, the insertion angle of the fibre affects the force. Concerning the force along the *y* axis, the maximum values measured were 2.99 × 10^−12^ *N *and 1.81 × 10^−12^ *N*, for 45° and 30°, respectively. From Fig. 11(a) one can also say, that the trap is quite symmetric. At 45° the PMMA particle reached a maximum velocity of 10.5 ± 0.7 µm/s, while moving towards the trapping point. This means that if the stage is moved with a constant velocity along *y* inferior to 10.5 µm/s, the particle will remain trapped. Regarding the force along the *x* direction, it is stronger along the +x direction than −x. This is due to the positive contribution of the axial force, pushing the particles towards the trap. When the particle is located beyond the trapping point, the optical force is weaker, reaching a maximum value of 8.02 × 10^−13^ *N*. In this case the particle reached a maximum velocity of 3.76 ± 0.54 µm/s. The graphs of Fig. 11 also show that both in transversal and axial directions, the optical trapping net force generally increases for higher tilting angles. The exception occurs along the direction of positive *x*, where the forces are very similar for both angles. This is likely related with the nature of the optical forces in this direction. In the direction of negative *x* the optical forces are the least intense, since the gradient force has to surpass the scattering, while in the direction of positive *x*, both scattering and gradient add positively to drive the particle to the trapping position. In this case, the contribution of the scattering and gradient components are possibly accountable for masking the effect of the tilt angle.

### Optical trapping of yeast cells

**Figure 12:**
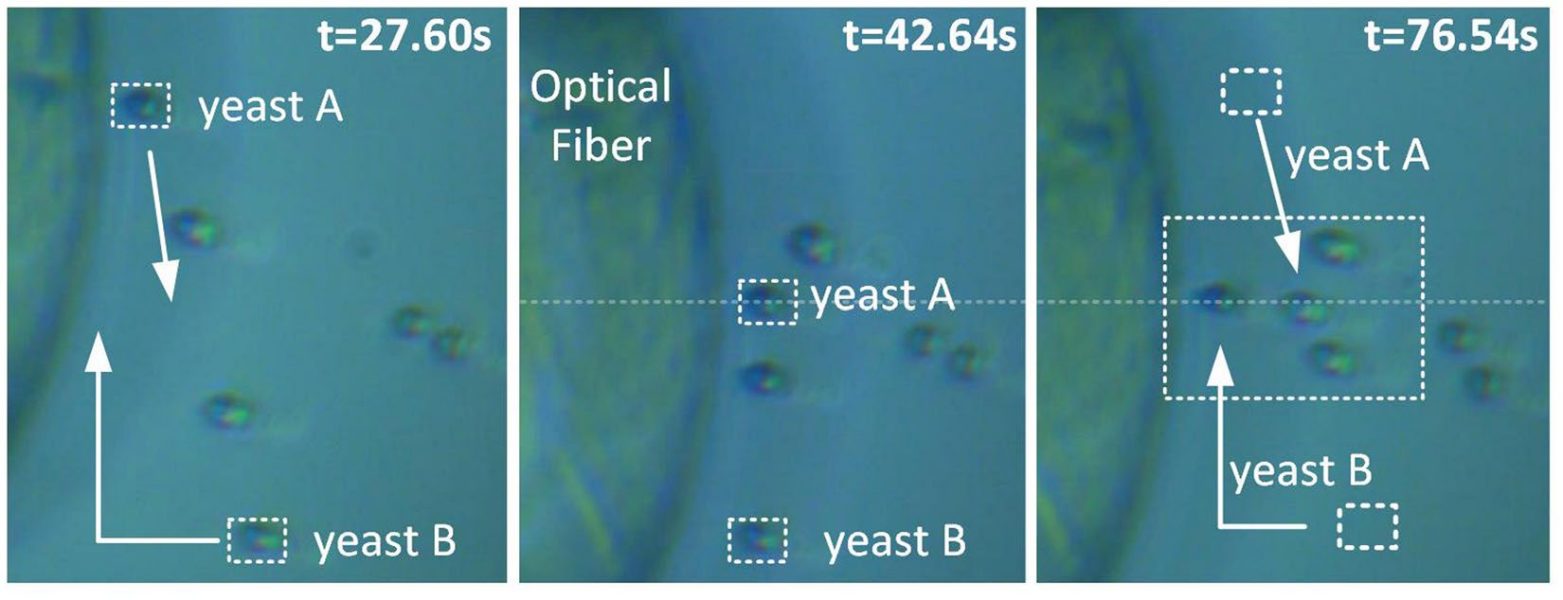
Rearrangement/sorting of two yeast cells, A and B, to a specific location (see Supplementary Video 2).

In a similar fashion, the trapping of yeast cells (refractive index of 1.49–1.53^50, 51^) was also tested using FPP-1. Keeping the fibre tilted at 45°, the yeasts could be stably trapped in 2D. The range of measured forces is in line with the previous calculations, reaching the maximum trapping force of 7.96 × 10^−13^ N experienced along *y* direction, and 3.36 × 10^−13^ N in *x* direction. The ability to trap in 2D was already verified with PMMA beads. Nevertheless, it is also important to demonstrate the capacity to move a specific target to an exact point. This is here demonstrated by Fig. 12. In this particular case, yeasts A and B, are separately driven to a defined point, indicated by the arrows. These end up aligned and between two other yeast cells. The full rearrangement of the yeasts can be seen in Supplementary Video 2.

### Detection of trapped particles

**Figure 13:**
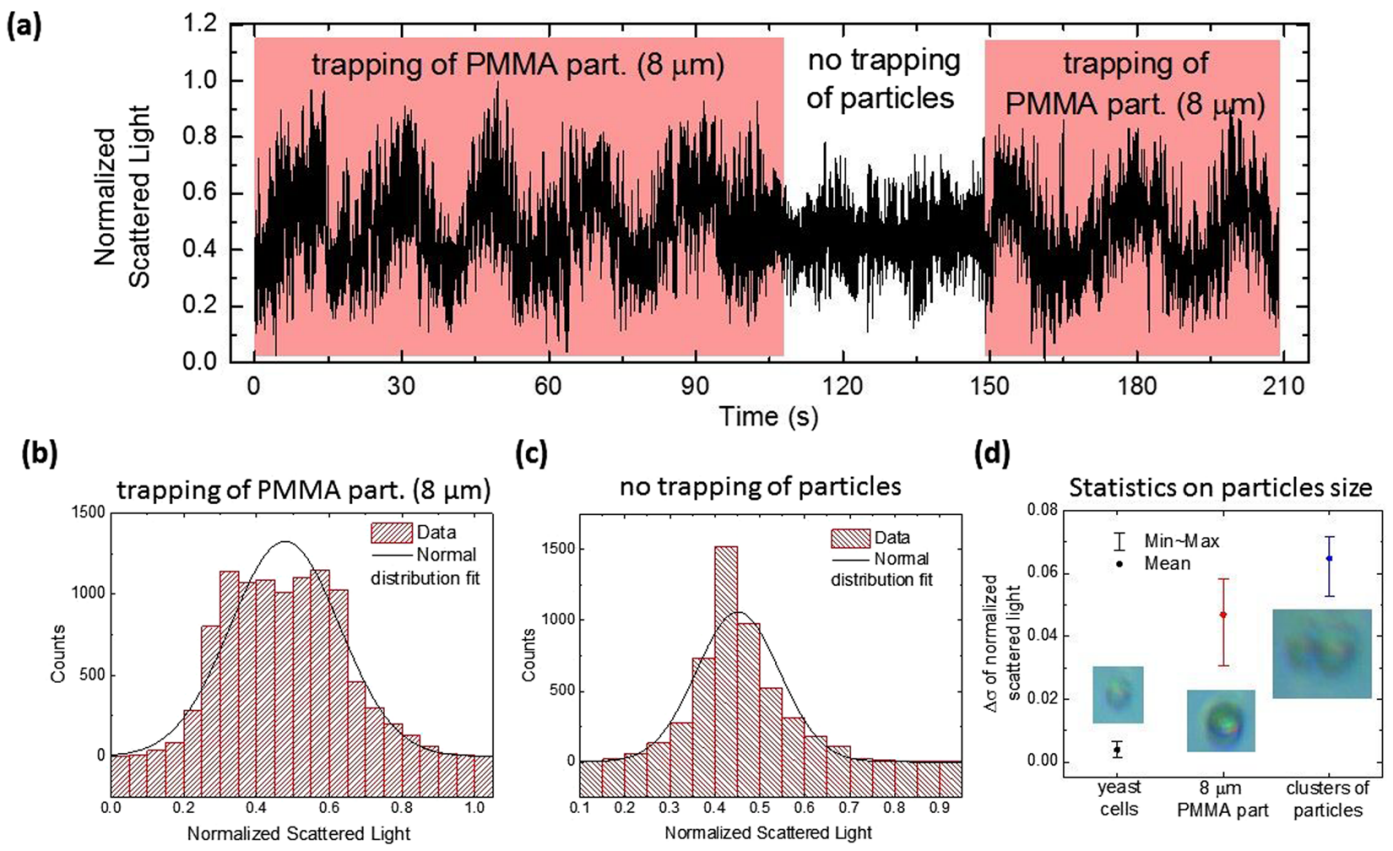
(**a**) Measurement of the light scattered by an 8 μm PMMA bead, and during the absence of trapping. (**b**) Normal distribution fit correspondent to the first trapping event shown in (**a**). (**c**) Normal distribution fit of the data representative of the absence of trapped particles. (**d**) Study of Δσ for yeast cells, 8 μm PMMA beads and clusters of particles. The images inside the graph are examples of the targets (scale photos).

With a slight modification of the setup it is straightforward to automatically detect and coarsely identify that a target particle is in the trapping area. Such particle detection system is composed by a photodetector (PDA 36A-EC, Thorlabs), connected to the optical fibre probe, using an optical fibre coupler. Thus, the laser can be injected in the probe and the backscattered light can be read by the PD. The 980 nm laser was modulated with a sinusoidal signal (frequency 1 MHz, amplitude 4 V) and the scattered light signal was then filtered with a second order Butterworth filter. An example of the acquired signal can be seen in Fig. 13(a). The presence or absence of an 8 µm bead near the trapping zone is made clear through the different response signals, as indicated in the graph. Acquiring multiple data for different targets (yeast cells, PMMA beads, and clusters of beads with a maximum size of 15 µm) allowed to identify significant differences in the scattered light, which could be the base of an identification system. To study this possibility, the data was statistically analysed, and each interval of information was fitted to a normal distribution. For every test, the standard deviation (σ) of the signal in the presence (*σ*_*trap*_) or absence (*σ*_*no trap*_) of targets, considered as the reference signal, was computed. Figure 13(b) shows the fit done to the first set of data (Fig. 13(a), trapping of a PMMA particle) while Fig. 13(c) show the fit in the absence of particle. Looking at these plots, one can see that *σ*_*no trap*_ should be smaller than *σ*_*trap*_. From the difference of these quantities, Δ*σ* = |*σ*_*no trap*_ − *σ*_*trap*_|, one can estimate ranges where particles of different sizes will belong to. This is depicted in Fig. 13(d). In this case, it is visible that yeast cells, with an average diameter of 4 to 5 µm are characterized by an average Δ*σ* of 0.00387, while for PMMA particles the value of Δ*σ* is 0.04686, and for clusters/aggregates of particles the value of Δ*σ* is 0.06473. These results show that larger particles do scatter more light and by this method it is possible to differentiate them.

## Discussion

The optical fibre probe allows to have full 2D trapping, allowing to push and pull dielectric particles as well as yeast cells. This unveils possible applications in biology related fields. Beyond this, the data gathered also proves that it is possible to select and arrange targets in a precise manner, which can be particularly useful in studies dedicated to single targets, where their isolation from the remaining sample population is required.

The lack of evidences of z trapping (3D), computational and experimentally verified, may be a constraint. However, for cell sorting and arrangement, the available capabilities hold great potential. To increase the trapping capabilities, as well as efficiency, further developments on the fabrication of the phase plates, that may lead to higher phase steps, is needed. In this case, the focal spot needs to be more confined, so that the electric field intensity gradient becomes steeper. According to Wright *et al*., beam spot sizes larger than 0.7 µm are not able to form 3D stable traps^52^. Such reduced spot sizes have recently been accomplished using binary phase plates fabricated by electron-beam lithography and dry etching. This validates the possibility to improve Fresnel plates using FIB milling, since both fabrication methods have resolutions of a few nanometers^53^.

The particle detection system, by analysis of the scattered light, enhances the applicability of the trapping setup, since sorting based on the specimen average size can be attained in an automatic fashion, if adequate feedback systems are implemented. With such approach, fibre tweezers enable immobilization, manipulation and detection all in the same platform, reducing the system cost and footprint, and greatly improving its potential applicability. Similar features, such as trapping and detection, have been recently reported by Yu-Chao Li *et al*., using nanojets to trap sub-wavelength targets^54, 55^. In this regard, this indicates the current trends on developing probes suitable for trapping but also having detection and sensing capabilities, that cover a wide range of scales from micro to nano.

## Conclusions

This paper reports the fabrication of Fresnel zone and phase plates by FIB milling on optical fibre tips. The design and fabrication of the structures was supported by computational simulations. In the case of the zone plates, the experimental data differs from the simulations. This reveals that the metallic rings might produce second order effects, that lead to the excitation of plasmons. Contrary to this, the phase plates experimental and computational data agree, demonstrating that this fabrication method allows to produce diffractive structures, that despite presenting some minimal error, still comply with the projections. With this in mind, the capacity of phase plates for trapping of cells and particles was tested and characterized. This is the first time, to our knowledge, that Fresnel phase plates fabricated by FIB milling on optical fibre tips are used to trap micrometric particles and cells. The use of such diffractive structures have been tested in the past for manipulation of sub-wavelength particles, but not for targets resembling mammalian cells. Beyond this, the optical fibre probe was simultaneously used to size-detect the particles.

To summarize, this paper explores new applications for Fresnel plates fabricated on optical fibre tips, beyond their common use as coupling devices. In the future, such devices may enable advanced monitoring and manipulation devices equipped with a feedback system, for automatic single particle/cell sorting according to their size.

## Electronic supplementary material

Supplementary Information

supplementary video 1

supplementary video 2
