# Supplementary material for: Fabrication of Fresnel plates on optical fibres by FIB milling for optical trapping, manipulation and detection of single cells
Source: Sci Rep. 2017 Jun 30;7:4485. doi: 10.1038/s41598-017-04490-2 (PMC5493682; doi:10.1038/s41598-017-04490-2)
Supplement: Supplementary file 1 — Supplementary Information [file 41598_2017_4490_MOESM1_ESM.pdf]

Supplementary Information

Fabrication of Fresnel plates on optical fibres by FIB milling for optical trapping, manipulation and detection of single cells

Rita S. Rodrigues Ribeiro,[1,2*] Pabitra Dahal,[2] Ariel Guerreiro,[1] Pedro A. S. Jorge[1] and Jaime Viegas[2]

[1] INESC TEC, Rua do Campo Alegre, 687, Porto, Portugal and Departamento de Física e Astronomia, Faculdade de Ciências, Universidade do Porto, Rua Campo Alegre, 687, Porto, Portugal
[2] Masdar Institute of Science and Technology, PO BOX 54224, Abu Dhabi, United Arab Emirates

*arsr@inescporto.pt

Video 1 Legend - Optical trapping of an 8 µm PMMA particle in the xy plane.

Video 2 Legend - Rearrangement/sorting of two yeast cells, A and B, to a specific location.
